# New dual-function *in situ* bone repair scaffolds promote osteogenesis and reduce infection

**DOI:** 10.1186/s13036-022-00302-y

**Published:** 2022-09-22

**Authors:** Changsheng Yang, Lei Zhou, Xiaodan Geng, Hui Zhang, Baolong Wang, Bin Ning

**Affiliations:** 1 Department of Orthopedic Oncology Surgery, Shandong University Cancer Center, Jinan, 250117 China; 2 Department of Orthopedic Oncology Surgery, Shandong Cancer Hospital and Institute Affiliated to Shandong First Medical University and Shandong Academy of Medical Science, Jinan, 250117 China; 3 Department of Radiology, Shandong Cancer Hospital and Institute Affiliated to Shandong First Medical University and Shandong Academy of Medical Science, Jinan, 250117 China; 4 Department of Medical Oncology, Shandong Cancer Hospital and Institute Affiliated to Shandong First Medical University and Shandong Academy of Medical Science, Jinan, 250117 China; 5 Department of Orthopedic Surgery, Shandong Provincial Third Hospital, Shandong University, Jinan, 250117 China; 6 Department of Orthopedic Surgery, Jinan Central Hospital, Shandong University, Jinan, 250117 China

**Keywords:** In situ, Dual-function, Infectious bone defect, Magnesium, Ampicillin

## Abstract

**Background:**

The treatment of infectious bone defects is a difficult problem to be solved in the clinic. In situ bone defect repair scaffolds with anti-infection and osteogenic abilities can effectively deal with infectious bone defects. In this study, an in situ polycaprolactone (PCL) scaffold containing ampicillin (Amp) and Mg microspheres was prepared by 3D printing technology.

**Results:**

Mg and Amp were evenly distributed in PCL scaffolds and could be released slowly to the surrounding defect sites with the degradation of scaffolds. *In vitro* experiments demonstrated that the PCL scaffold containing Mg and Amp (PCL@Mg/Amp) demonstrated good cell adhesion and proliferation. The osteogenic genes *collagen I (COL-I)* and *Runx2* were upregulated in cells grown on the PCL@Mg/Amp scaffold. The PCL@Mg/Amp scaffold also demonstrated excellent antibacterial ability against *E. coli* and *S. aureus*. *In vivo* experiments showed that the PCL@Mg/Amp scaffold had the strongest ability to promote tibial defect repair in rats compared with the other groups of scaffolds.

**Conclusions:**

This kind of dual-function in situ bone repair scaffold with anti-infection and osteogenic abilities has good application prospects in the field of treating infectious bone defects.

## Introduction

Infectious bone defects due to traffic accidents, sports competition, war, and industrial production activities are the main causes of postoperative infections [1]. Severe infectious bone defects and irregular surgical procedures can cause a large number of bacteria to invade and multiply at the wound site, eventually leading to severe osteomyelitis [2]. Severe osteomyelitis can lead to reduced quality of life, disability, and amputation of limbs [3]. If the treatment of osteomyelitis is not timely, it will develop into chronic osteomyelitis. Chronic osteomyelitis will increase the difficulty of treatment, imposing a huge economic burden on patients and society [4]. Therefore, patients with infectious bone defects require both bone repair and anti-infection treatment to ultimately achieve full functional recovery of the bone injury.

Treatment of infectious bone defects includes removal of necrotic bone fragments, local and systemic administration of antibiotics, and repair of the bone defect with bone grafts [5]. Autologous bone grafts are considered the “gold standard” for the treatment of bone defects due to their excellent osteoinductive ability [6]. However, autologous bone grafts are a limited source and can cause morbidity in donor sites, so they are gradually being replaced by tissue engineered scaffolds. In 1993, professor Robert Langer of Massachusetts Institute of Technology first proposed the concept of “Tissue Engineering” [7]. Tissue engineering is the combination of biotechnology and engineering to develop functional substitutes for the repair of diseased and incapacitated tissues and organs. Tissue engineering technology is an interdisciplinary field that mainly involves life science and engineering technology. Tissue engineering consists of three elements: cells, growth factors and scaffolds [8]. Although bone tissue engineering scaffolds with cells have excellent bone repair ability, such scaffolds also have many disadvantages, such as limited cell sources, complex scaffold preparation processes and immunogenicity [9]. To solve the problems existing in traditional tissue engineering technology, a new concept, “in situ tissue engineering”, was proposed and developed [10, 11]. In situ tissue engineering, scaffolds do not carry cells but instead use the physical, chemical, and biological properties of scaffolds to recruit stem cells to promote the bone repair process [12].

In situ tissue engineering scaffolds are cell-free and belong to the next generation of tissue engineering scaffolds. Scaffolds are designed to achieve the functions of immune regulation, vascularization, stem cell recruitment and osteogenic differentiation *in vivo* through physical, chemical and biological functionalization [13]. The physical characteristics of the scaffold mainly include the surface morphology of the pore size [14, 15]. Appropriate physical characteristics are favorable for cell adhesion, cell migration and cell differentiation. The chemical properties of scaffolds are mainly determined by the end groups and surface charge of the materials, which determine the hydrophilicity and hydrophobicity of the surface [15, 16]. The biological characteristics of scaffolds are mainly regulated by bioactive molecules in scaffolds. These bioactive molecules mainly include drugs [17], growth factors [18], peptides [19, 20], rare metals [21], microRNA (miRNA) [22], chemokines [23] and so on. Their functions are mainly to recruit stem cells and promote vascularization and osteogenic differentiation.

To treat infectious bone defects caused by open bone trauma, many research teams have developed in situ bone repair scaffolds with both antibacterial and osteoinductive properties [24]. Dongyun Wang et al. synthesized biomimetic calcium phosphate (BioCaP) containing BMP2 and hydroxypropyl trimethyl ammonium chitosan (HACC) materials. The bone morphogenic protein (BMP2) in the material has the function of promoting osteogenesis, and HACC has an antibacterial ability against methicillin-resistant *Staphylococcus aureus* (MRSA). The *in vivo* results showed that the density of newly formed bone in the HACC (0.8 μg) + BMP2 and HACC (4 μg) + BMP2 groups was higher than that in the BMP2 group. These results suggest that the antibacterial ingredient HACC can enhance the osteogenic ability of BMP2-BioCaP material by inhibiting *in vivo* infection. Lei Chen et al. prepared PLGA bone repair scaffolds using fused deposition modelling (FDM) 3D printing technology and immobilized BMP2 and antimicrobial peptides on the surface of scaffolds using polydopamine (pDA) [25]. BMP2 and antimicrobial peptides on the surface of the scaffold showed slow release within 72 h. *In vitro* experiments showed that the scaffolds exhibited good antibacterial properties and promoted osteogenic differentiation.

In this study, an in situ bone scaffold for treating infected bone defects was prepared using FDM 3D printing technology. Ampicillin (Amp) and magnesium (Mg) microspheres were added to the polycaprolactone (PCL) material to prepare the composite bone scaffold. Amp and Mg microspheres could be slowly released to surrounding tissues as PCL degraded. Amp had a broad spectrum of bactericidal ability against gram-negative and gram-positive bacteria. Mg microspheres could release Mg^2+^ and promote vascularization and osteogenic differentiation of cells. This kind of dual-function in situ bone repair scaffold can be used to treat infectious bone defects.

## Materials and methods

### Materials

PCL (Mn = 80000) was purchased from Shanghai Aladdin Biochemical Technology Co., Ltd. (China). Calcein AM reagent, CCK-8 Kit and ampicillin (C_16_H_19_N_3_O_4_S, Mw = 349.4) were purchased from Beijing Solarbio Co., Ltd. (China). Mg microspheres (diameter is 10 ~ 60 µm) were purchased from Chengdu Haoming Technology Co., Ltd. (China). TRIzol reagent was purchased from Invitrogen (USA). The PrimeScript™ RT Master Mix Kit and TB Green® Fast qPCR Mix Kit were purchased from Beijing Takara Biomedical Technology Co., Ltd. (Japan). *q*PCR primers were synthesized by Jilin Comate Bioscience Co., Ltd. (China).

### 3D print scaffolds

Mg microspheres and AMP were added to the PCL materials and mixed using a banbury mixer (Hartek, China) at 80 °C for 10 min. The contents of Mg, AMP and PCL in the different groups are shown in Table 1. The mixed composite material was cooled and cut into particles for subsequent scaffold printing. Solidworks2019 software was used to design the appearance size of the scaffolds (22 mm × 22 mm × 2.5 mm). The scaffold was printed using an FDM 3D printer (Ubbiotech Co., Ltd.). The printing speed was 20 mm/s, and the printing temperature was 80 °C. The appearance of the scaffolds was photographed with a digital camera (D850, Nikon). The scaffold was scanned by micro-CT (Scansky1172, Bruker).

**Table 1:** Weight percentage of Mg, Amp and PCL in different groups

Group name	PCL (%)	Amp (%)	Mg (%)
PCL	100	-	-
PCL@Amp	95	5	-
PCL@Mg	90	-	10
PCL@Mg/Amp	85	5	10

### Scaffold characterization

The morphology of the surface and cross section of different scaffolds were observed by a field emission scanning electron microscope (SEM, Gemini 2, Zeiss). The diameter distribution of Mg microspheres was analysed by ImageJ software based on the SEM images. The surface functional groups were identified using a Fourier transform infrared spectrometer (FTIR, TENSOR 27, Bruker). The chemical components of the scaffolds were characterized by TGA (Q500, TA) and XRD (D8 Advance, Bruker). The contact angle was measured by a contact angle meter (DSA100E, KRUSS). The 200 mg scaffold was immersed in 2 ml of deionized water and incubated on a shaker at 80 rpm and 37 ℃. The concentrations of Mg^2+^ released from the scaffold at 3, 9, 15 and 21 days were measured using inductively coupled plasma–mass spectrometry (ICPMS, Varian, Darmstadt, Germany).

### Cell adhesion and proliferation

MC-3T3-E1 cells were used to evaluate cell adhesion and proliferation on the scaffold surface. The cell culture medium contained DMEM (Gibco) with 10% FBS (Gibco), 1% penicillin and 1% streptomycin (Sigma). The culture conditions were 37 °C with 5% CO_2_ in a humidified incubator. After two passages, the cells were seeded on the surface of the scaffold at a density of 5 × 10^4^ cells/ml. The CCK-8 Kit was used to detect cell proliferation on days 1 and 3 according to the instructions. The OD450 value was measured by a plate reader (M200, TECAN). On day 3, live and dead cells were stained using Calcein AM and PI according to the instructions. The cells were photographed using an inverted fluorescence microscope (TE2000U, Nikon).

### Osteogenesis differentiation

The expression levels of *collagen I (COL-I*) and *Runx2* were used to evaluate osteogenic differentiation. As shown in Table 2, *q*PCR primers for *COL-I* and *Runx2* were synthesized according to previous literature [26]. MC-3T3-E1 cells were cultured on different scaffold surfaces as described in the cell adhesion and proliferation section. After 3 days of cell culture, TRIzol was used to extract total RNA from the cells. Total RNA concentration and purity were measured using a Nanodrop plate (M200, TECAN). According to the instructions, 500 ng of total RNA was reverse-transcribed into cDNA using the PrimeScript™ RT Master Mix Kit. The relative expression levels of *COL-I* and *Runx2* were quantified using TB Green® Fast qPCR Mix Kit according to the manufacturer’s protocol.

**Table 2:** List of the qPCR primers for *COL-I* and *Runx2*

Gene Annotation	Primer Sequence (5’-3’)
*COL-I*	F: CGCTGGCAAGAATGGCGATC
	R: ATGCCTCTGTCACCTTGTTCG
*Runx2*	F: GCCCTCATCCTTCACTCCAAG
	R: GGTCAGTCAGTGCCTTTCCTC

### Scaffold mineralization ability

All groups of scaffolds were immersed in centrifuge tubes containing 10 ml of simulated body fluid. These centrifuge tubes were incubated in a 50 rpm shaker at 37℃. The simulated fluid in the centrifuge tube was changed daily. At 24 days, the surface morphology of the scaffold was evaluated by SEM.

### Antibacterial ability

*Staphylococcus aureus* (*S. aureus*) and *Escherichia coli* (*E. coli*) were used to evaluate the antibacterial activity of the scaffolds. The strains were cultured in LB medium at 37 °C and 150 rpm. Five hundred microliters of 4.0 × 10^4^ bacteria/ml were spread on an LB agar plate and then dried on an ultraclean workbench. The scaffolds of different groups were placed in agar plates and cultured upside down overnight in a 37 °C incubator. A digital camera (D850, Nikon) was used to photograph the bacteriostatic ring on the agar plate. The diameter of the bacteriostatic ring was measured by ImageJ software.

### In situ repair of tibial defects in rats

A rat model of tibial open bone defects was used to evaluate the bone repair ability of the scaffold. Twelve male SD rats weighing approximately 180 g were divided into the PCL, PCL@Amp, PCL@Mg and PCL@Mg/Amp groups. Pentobarbital sodium (3.5%, 1 ml/kg) was used to anesthetize the rats by intraperitoneal injection. An electric shaver was used to remove hair from the hind legs of the rats. A scalpel was used to cut the skin and expose the tibia. The tibia was drilled with a 2.5 mm diameter bone-taking drill. Then, different groups of scaffolds were cut into appropriate sizes and implanted into the defect. Finally, the wound was sutured with surgical thread. Eight weeks after surgery, all rats were sacrificed, and bone defects were scanned by micro-CT (Scansky1172, Bruker). CtAn software (Bruker) was used to calculate the BV/TV% of the defect location. All bone samples were decalcified, followed by H&E and Masson staining to analyse the effect of bone repair at the histological level. All animal experimental protocols were carefully checked and approved by Shandong Cancer Hospital and Institute Affiliated to Shandong First Medical University.

### Statistical analysis

All data are shown as the means ± standard deviation. One-way analysis of variance with a post hoc test was used to determine significant differences by Origin 2017. *p* < 0.05 represents a statistically significant difference. and the data are indicated with (*) for probability less than 0.05 (*p* < 0.05).

## Results and discussion

### Scaffold characterizations

**Fig. 1:**
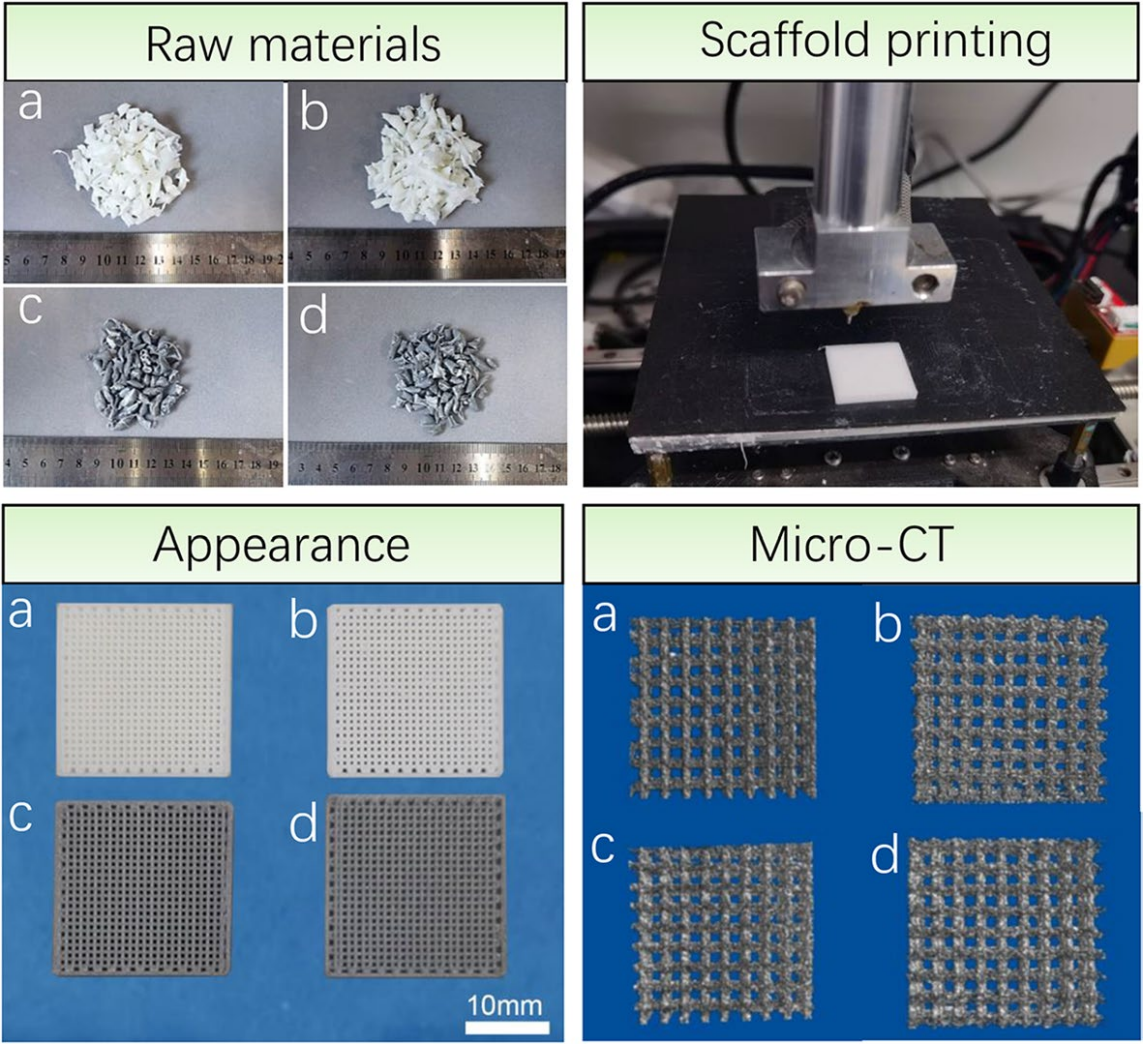
Composite raw material particles, scaffold printing, macro appearance and micro-CT images. (a) PCL, (b) PCL@Amp, (c) PCL@Mg and (d) PCL@Mg/Amp

As shown in Fig. 1, the different composite raw materials were processed into particles by the banbury mixer. The composite raw materials PCL@Mg and PCL@Mg/Amp appeared gray. PCL and PCL@Amp appeared inherently white. These raw materials were printed by an FDM printer into scaffolds at 80 °C. The macro appearance size of the scaffold was basically consistent with the software design. Micro-CT images of scaffolds showed an interconnected pore structure. The above results showed that PCL mixed with Mg and Amp did not affect the whole printing process.

**Fig. 2:**
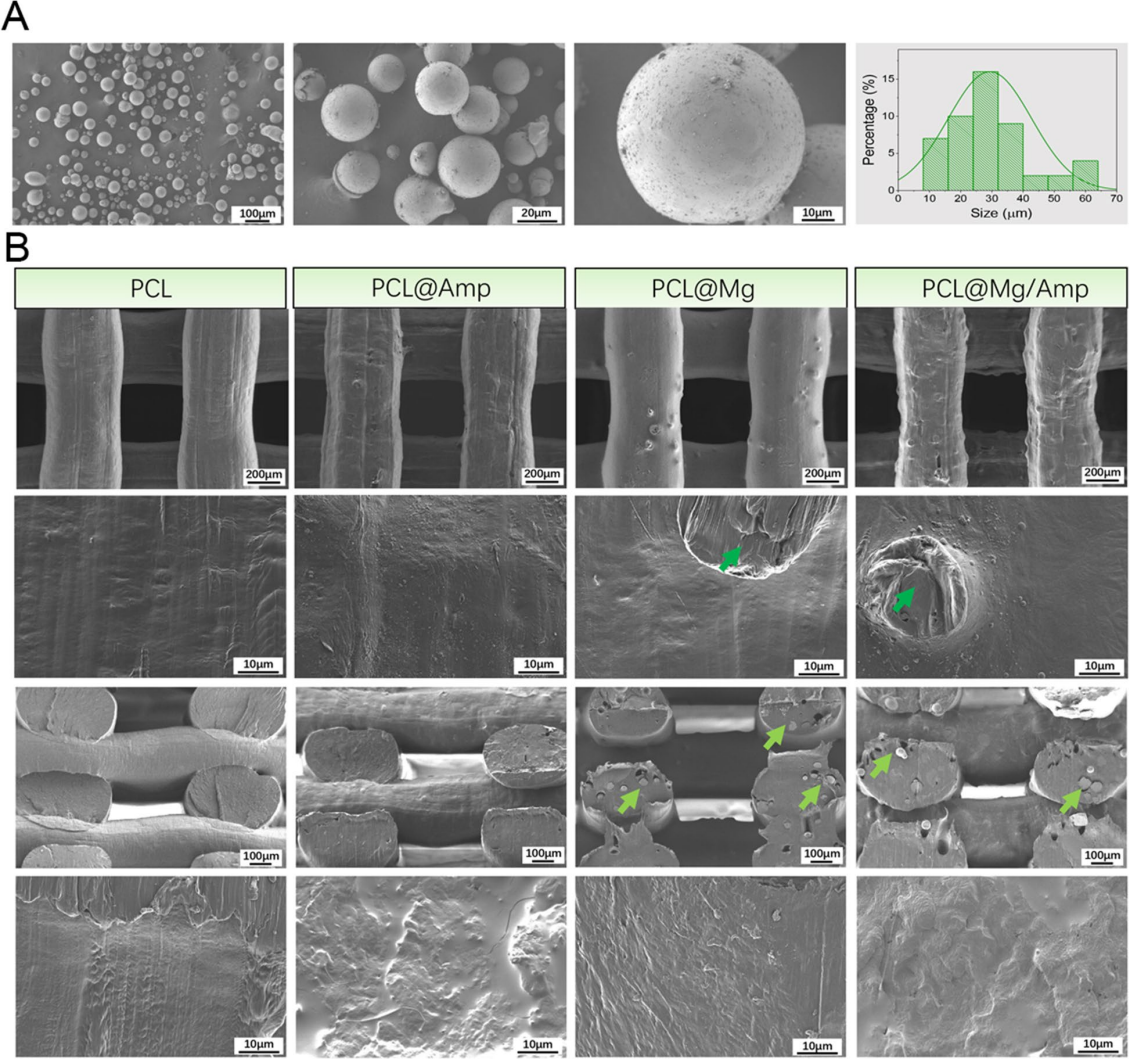
**A** SEM images of Mg microparticles and diameter distribution. **B** SEM images of the surface and cross-section morphology of the scaffold. The green arrow indicates the Mg microsphere

As shown in Fig. 2A, Mg had a spherical structure, and the microspheres were 10—60 μm in diameter. The average diameter of the Mg microspheres was 29.38 μm. As shown in Fig. 2B, SEM images show the morphology of the surface and cross section of the scaffold. The filament diameter of the scaffold was 436 ± 7.2 μm, and the pore size of the scaffold was 420.66 ± 12.1 μm. The filaments and pores of the scaffolds were very uniform and regular. The scaffold surface of the PCL and PCL@Mg groups was relatively smooth. The surface of the PCL@Amp and PCL@Mg/Amp groups became slightly rough due to the addition of Amp powder. In the PCL@Mg and PCL@Mg/Amp groups, Mg microspheres were embedded on the surface of the scaffold filaments. SEM images of the cross section of the scaffold showed that Mg microspheres in the PCL@Mg and PCL@Mg/Amp groups were evenly dispersed inside the scaffold filaments.

**Fig. 3:**
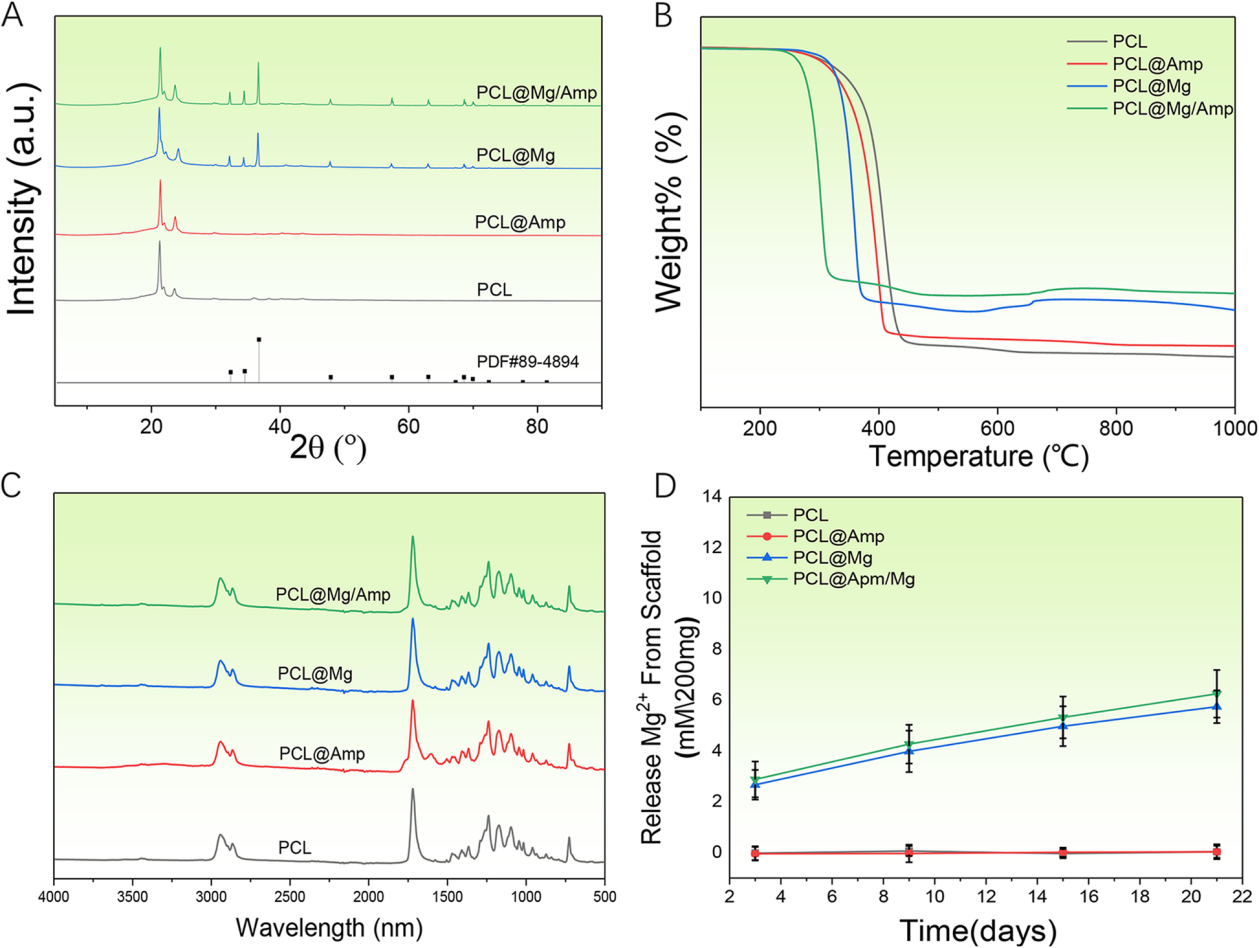
Scaffold characterizations: (**A**) XRD, (**B**) TGA, (**C**) FTIR patterns and (**D**) Mg^2+^ release from scaffolds

**Fig. 4:**
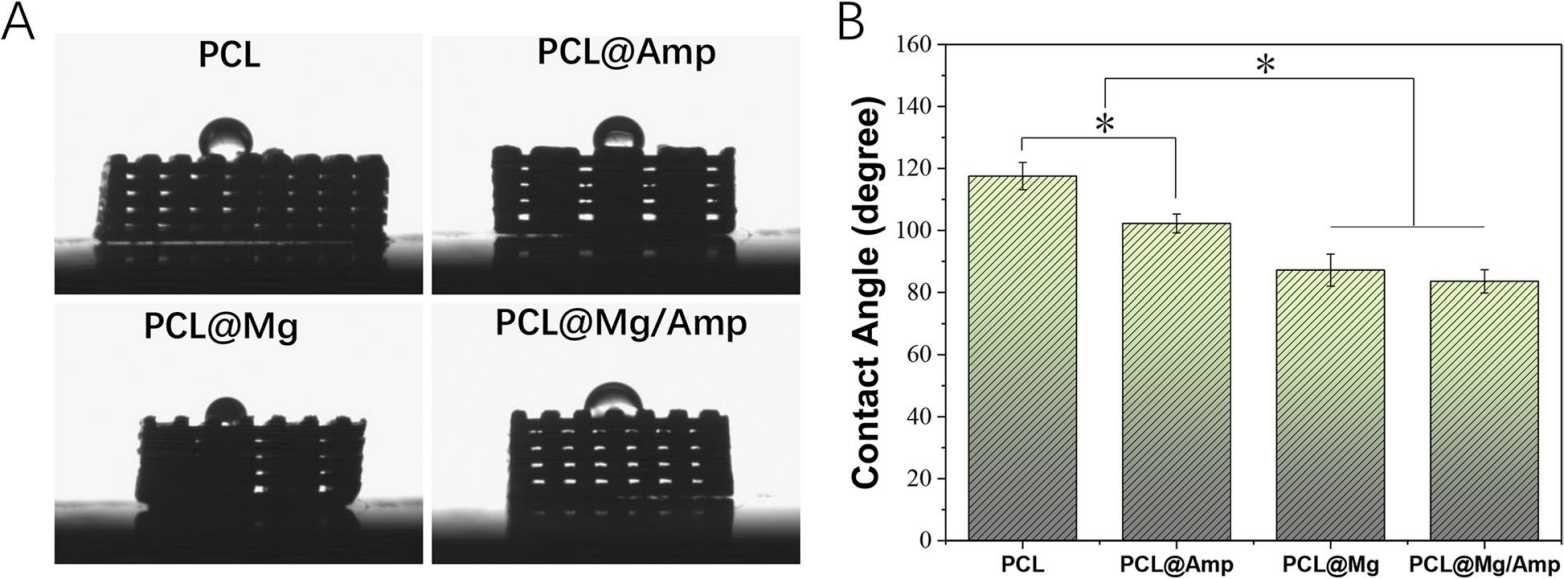
Measurement of the contact angle of different scaffolds, *n* = 3 *p* < 0.05

XRD, TGA and FTIR were used to determine the composition of the scaffold. The phase composition was checked in accordance with the Joint Committee on Powder Diffraction Standards reference patterns of Mg (PDF No. 89–4894). With the addition of Mg microparticles to the PCL@Mg/Amp and PCL@Mg scaffolds, the diffraction pattern included several peaks caused by Mg microparticles (Fig. 3A). Amp showed an absorption peak in the region of 1730 – 1720 cm^−1^, which is caused by C = O β-lactam stretching. The peak at 1610 cm^−1^ belongs to C = O amide stretching [27] (Fig. 3B). TGA was used to analyse the contents (W/W) of Mg and Amp in different scaffolds. PCL in the scaffold began to decompose at 225 °C. The mass losses of PCL, PCL@Amp, PCL@Mg and PCL@Mg/Amp were 99.902%, 94.221%, and 83.482%, respectively (Fig. 3C). The PCL@Mg and PCL@Mg/Amp scaffolds showed a slow release of Mg^2+^ profile during the whole 21-day release period, while the scaffolds in the PCL and PCL@Amp groups did not release Mg^2+^. At 3 days, the cumulative release of Mg^2+^ in the PCL@Mg and PCL@Mg/Amp groups was 2.67 ± 0.58 mM and 2.89 ± 0.7 mM, respectively. At 21 days, the cumulative release of Mg^2+^ in the PCL@Mg and PCL@Mg/Amp groups was 5.75 ± 0.64 mM and 6.26 ± 0.94 mM, respectively (Fig. 3D). As shown in Fig. 4, the water contact angles of different groups of scaffolds were measured to assess the surface hydrophilicity and hydrophobicity. The contact angles of the PCL, PCL@Amp, PCL@Mg and PCL@Mg/Amp groups were 117.5 ± 4.4°, 102.2 ± 3.0°, 87.2 ± 5.2° and 83.5 ± 3.7°, respectively. PCL showed an obvious hydrophobic surface, which was consistent with previous literature [28]. With the addition of Amp and Mg, the hydrophilicity of the PCL composite scaffold surface was significantly enhanced.

3D printing technology can be used to personalize and prepare various macro morphologies of bone repair scaffolds. In 1983, Chuck Hull et al. invented stereo lithography appearance (SLA) 3D printing technology [29], and in 1988, S. Scott Crump invented FDM 3D printing technology [30]. FDM 3D printing technology completes the whole printing process of the scaffold by melting the polymer material at high temperature and then extruding, printing and cooling. The printing process of FDM does not require the addition of photoinitiator (LAP) included in SLA printing technology. Previous literature has shown that LAP is obviously cytotoxic, which decreases the bone repair ability of scaffolds [31]. Based on the above reasons, the bone repair scaffold prepared in this study uses FDM printing technology. Currently, polylactic acid (PLA), poly(lactide-co-glycolide) (PLGA), PCL and other polymer materials are commonly used in the preparation of bone repair scaffolds. The melting point of PCL is 58 – 64 °C, which can be used to mix some drug components with poor thermal stability. In this study, PCL scaffolds containing Mg and AMP were successfully printed at 80 °C. SEM images showed that Amp and Mg were evenly mixed in the PCL material, which could be slowly released into surrounding tissues with the degradation of PCL *in vivo* (Fig. 2B). Compared with surface coating technology, Amp and Mg^2+^ were released slowly throughout scaffold degradation and new bone regeneration^25^. TGA, XRD and FTIR results showed that Mg and Amp were successfully mixed into the scaffold without any change in the phase pattern and molecular structure (Fig. 3). The contact angle of the scaffold surface has an important effect on cell adhesion and proliferation^15^. The surface with a 65° contact angle was the most favourable for osteoblast adhesion, while both high and low contact angles were unfavourable for osteoblast adhesion. The contact angle of the PCL@Mg/Amp group was 83.5 ± 3.7°, which was the closest scaffold to 65°, and it was speculated that it was the most conducive to cell adhesion and proliferation among all scaffolds. PCL is a biodegradable polyester approved by Food and Drug Administration (FDA) in tissue engineering applications [32]. PCL degrades more slowly than PLA and PLGA *in vivo* [33]. The molecular weight of PCL decreased gradually within 2 years, and degraded to fragments of low molecular weight after 2 years [34].The increased hydrophilicity of the scaffold can increase the rate of water diffusion into the PCL polymer, which may also increase the degradation rate of the scaffold [34].

### Cytocompatibility

**Fig. 5:**
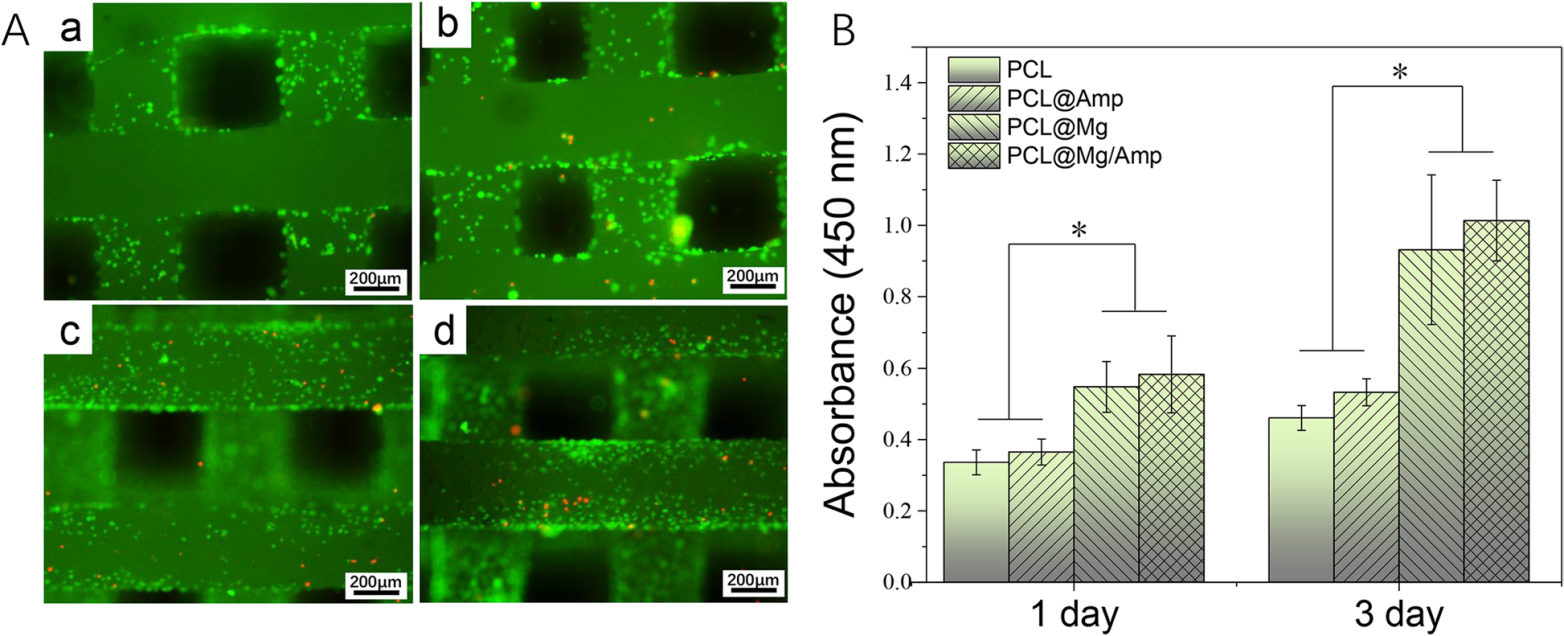
**A** Calcium AM staining of adherent cells on scaffolds and (**B**) CCK-8 assay of cell proliferation, *n* = 3 *p* < 0.05

As shown in Fig. 5A, the cell adhesion and proliferation abilities were measured at 1 and 3 days. The amount of cell adhesion was relatively low in the PCL and PCL@Amp groups. In contrast, there was a significant increase in cell adhesion in the PCL@Mg and PCL@Mg/Amp groups. In all groups, very few dead cells were present. As shown in Fig. 5B, the proliferation ability of the PCL@Mg and PCL@Mg/Amp groups was significantly higher than that of the PCL and PCL@Amp groups at 1 and 3 days. The proliferation ability of the PCL@Amp group was slightly higher than that of the PCL group, and the same trend was slightly higher in the PCL@Mg/Amp group than in the PCL@Mg group.

As shown in Fig. 4, the addition of AMP and Mg microspheres significantly improved the hydrophilicity of the scaffold, and the cell adhesion and proliferation results were consistent with the contact angle results. Since the contact angle of the PCL@Mg/Amp group was closest to 65°, its cell adhesion and proliferation were best [15]. Previous literature has also demonstrated that Mg^2+ is^ involved in platelet-derived growth factor (PDGF)-stimulated MC-3T3-E1 cell adhesion and proliferation [35]. The Mg2 + concentration is also important for cell adhesion and proliferation. Jie Shena et al. found that a Mg^2+^ concentration of approximately 4.11 mM was most conducive to cell adhesion and proliferation of MC-3T3-E1 cells. The cumulative release concentration of Mg^2+^ ranged from 2–7 mM in the PLC@Mg and PCL@Mg/Amp groups during the 3–21 days release period, which were all within the appropriate concentration range that could promote cell adhesion and proliferation (Fig. 3D). PDGF is generally believed to have the ability to promote the adhesion and proliferation of many cell types. Therefore, the PCL@Mg and PCL@Mg/Amp groups with bioactive molecular Mg significantly promoted cell adhesion and proliferation.

### Expression of osteogenic genes and mineralization ability

**Fig. 6:**
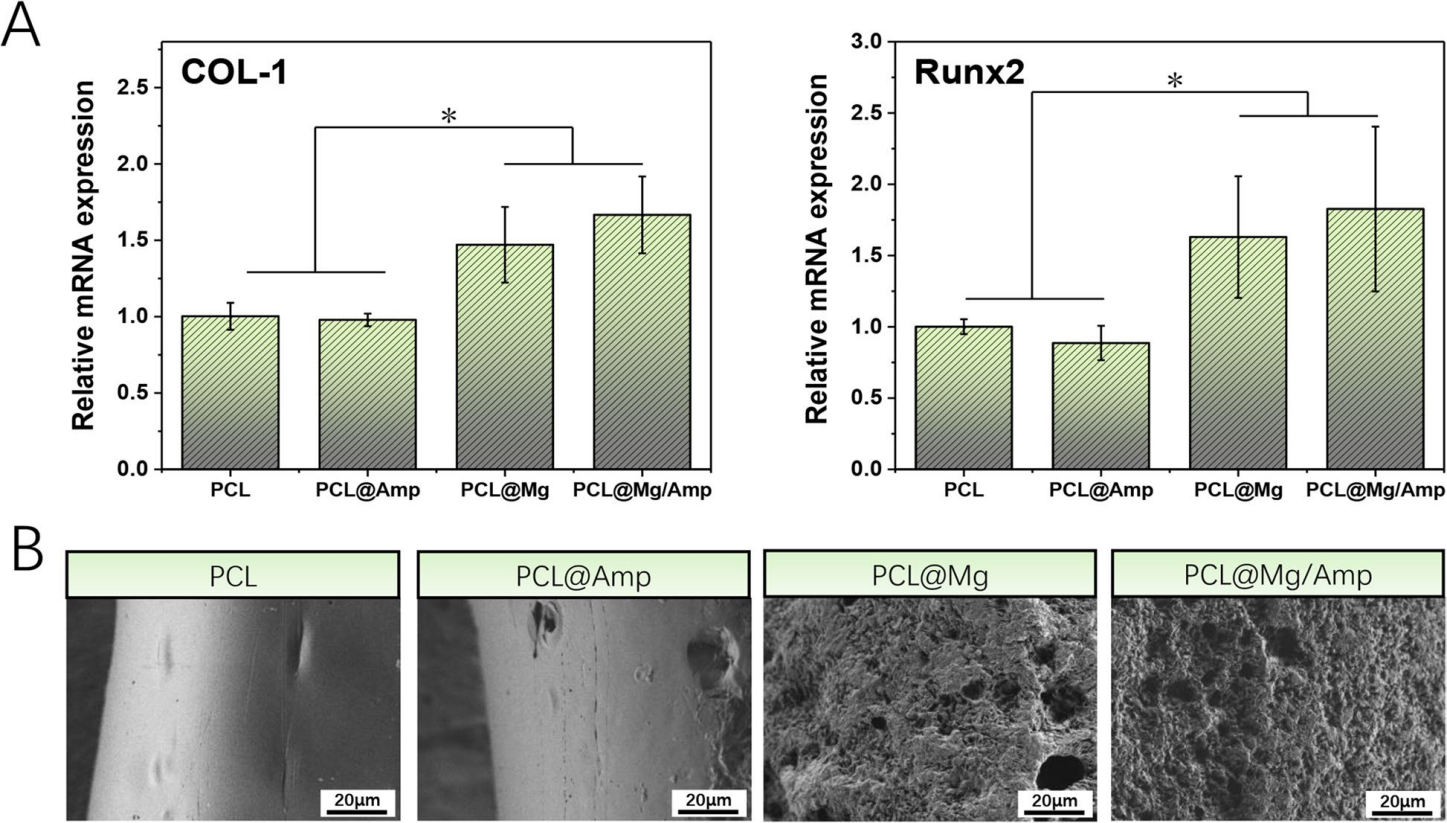
**A** The relative mRNA expression of *COL-I* and *Runx2*, *n* = 3 *p* < 0.05. **B** The mineralization ability of different scaffolds evaluated by SEM

As shown in Fig. 6A, the *COL-I* and *Runx2* genes were selected to evaluate the osteogenic differentiation ability of scaffolds at 3 days. *q*PCR results showed that the mRNA expression levels of *COL-I* and *Runx2* in PCL@Mg and PCL@Mg/Amp scaffolds containing Mg were significantly higher than those in PCL and PCL@Amp scaffolds without Mg. The *COL-I* expression levels in the PCL@Mg and PCL@Mg/Amp groups were 1.47 and 1.67 times higher than that in the PCL group, respectively. *Runx2* expression levels were 1.63 and 1.83 times higher in the PCL@Mg and PCL@Mg/Amp groups than in the PCL group, respectively. As shown in Fig. 3D, the Mg-containing scaffold prepared in this study can slowly release Mg^2+^ into the surrounding solution, which is mainly attributed to the reaction of Mg with water to generate Mg^2+^, hydrogen and OH^−36^. Many previous studies have confirmed that Mg^2+^ can promote osteogenic differentiation through upregulation of osteogenic gene expression, alkaline phosphatase (ALP) secretion and mineralization [36–39]. Qiangsheng Dong et al. used FDM 3D printing technology to fabricate Mg-containing PCL scaffolds. *q*PCR results showed that the osteogenic genes *osteopontin (OPN), osteocalcin (OCN)*, *COL-I* and *Runx2* were significantly upregulated during 7–21 days of culture [36]. Researchers have found that Mg^2+^ mainly regulates osteogenic differentiation through the PI3 K/Akt signaling pathway [40]. Mineralization capacity is also an important item to evaluate the osteogenesis of in-situ bone repair scaffolds. As shown in Fig. 6B, the surface of the scaffolds of PCL and PCL@Amp groups were relatively smooth, and the effect of mineralized deposition was not obvious. In contrast, the PCL@Mg and PCL@Mg/Amp groups had a rough and uneven surface, which was attributed to the surface mineralization of the scaffold. Degradation of Mg microspheres in scaffolds will release Mg^2+^ and form an alkaline environment, which will promote the formation of apatite [41]. The scaffolds containing Mg microspheres showed obviously mineralization ability, which was consistent with the results of the relative mRNA expression of *COL-I* and *Runx2* genes.

### Antibacterial ability

**Fig. 7:**
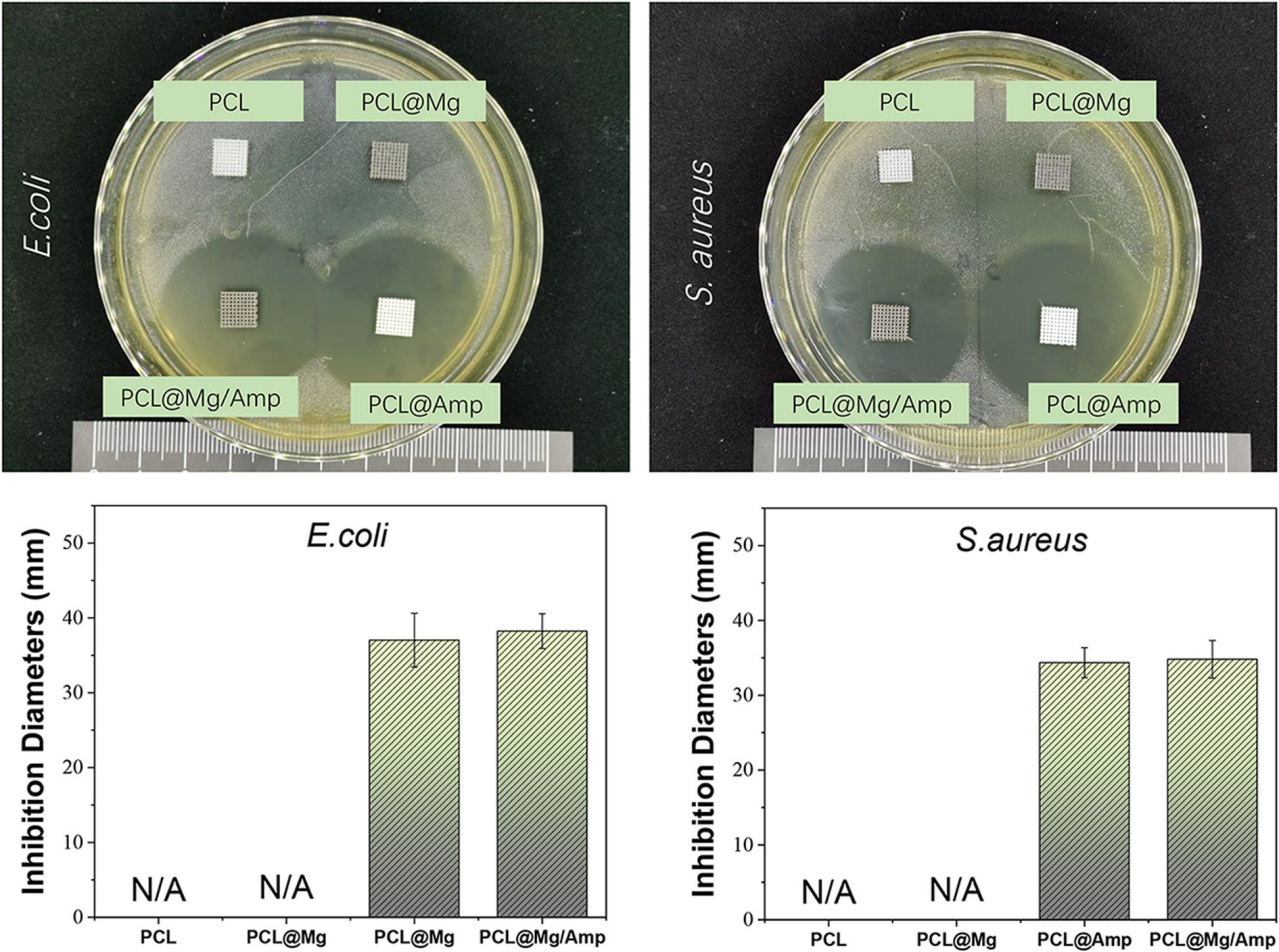
Antibacterial ability of different groups of scaffolds against *E. coli* and *S. aureus*, *n* = 3 *p* < 0.05

As shown in Fig. 7, the bacteriostatic ring test against *E. coli* and *S. aureus* was used to evaluate the antibacterial ability of the scaffolds. There was no obvious bacteriostatic ring *E. coli* or *S. aureus* around the PCL and PCL@Mg scaffolds. In contrast, the Amp-containing scaffolds PCL@Amp and PCL@Mg/Amp showed obvious bacteriostatic rings against *E. coli* and *S. aureus.* The diameters of bacteriostatic rings formed by PCL@Amp and PCL@Mg/Amp on the surface of agar plates coated with *E. coli* were 37.0 ± 3.6 mm and 38.2 ± 2.3 mm, respectively. The diameters of bacteriostatic rings formed by PCL@Amp and PCL@Mg/Amp on the surface of agar plates coated with *S. aureus* were 34.3 ± 2.0 mm and 34.8 ± 2.5 mm, respectively.

Antibacterial agents used to inhibit open bone trauma mainly include antimicrobial peptides, silver nanoparticles (AgNPs), antibiotics, etc. Lei Chen et al. used pDA molecules to attach antimicrobial peptides (ponericin G1) to the surface of PLGA scaffolds, and ponericin G1 showed good antibacterial effects on *E. coli* and *S. aureus* [25]. The advantage of antimicrobial peptides is that it is not easy to develop antimicrobial resistance, and they can have broad-spectrum antimicrobial activity [42]. However, antimicrobial peptides are composed of amino acids, and their thermal stability is relatively poor, so they cannot be directly used in FDM 3D printing. AgNPs with diameters between 1–100 nm have been widely used in the preparation of antibacterial scaffolds due to their strong antibacterial activity [43]. Jiayi Li et al. used FDM 3D printing technology to prepare a PCL bone repair scaffold with AgNP coating on the surface, which showed a significant bacteriostatic effect on *S. aureus. In vivo* animal experimental results showed that scaffolds containing AgNP coatings had the best effect on repairing infected bone defects at the external tibial epicondyle of rabbits [44]. Antibiotics such as vancomycin, tobramycin, tetracyclines, and Amp have been used to treat bone defects [24]. The pharmacological mechanisms of these antibiotics have been well studied and approved by regulatory authorities, so their safety *in vivo* was higher than that of other antibacterial agents [45]. Antibiotics also have higher thermal stability than antimicrobial peptides, so scaffolds containing antibiotics can be prepared using FDM 3D printing technology. Amp, the most commonly used antibiotic in clinical practice, can be used to treat osteomyelitis [46]. In this study, Amp was used as an antibacterial agent to treat bone defects. The PCL scaffold can also be mixed with other types of antibiotics to treat different bacterial infections.

### *In vivo* bone regeneration

**Fig. 8:**
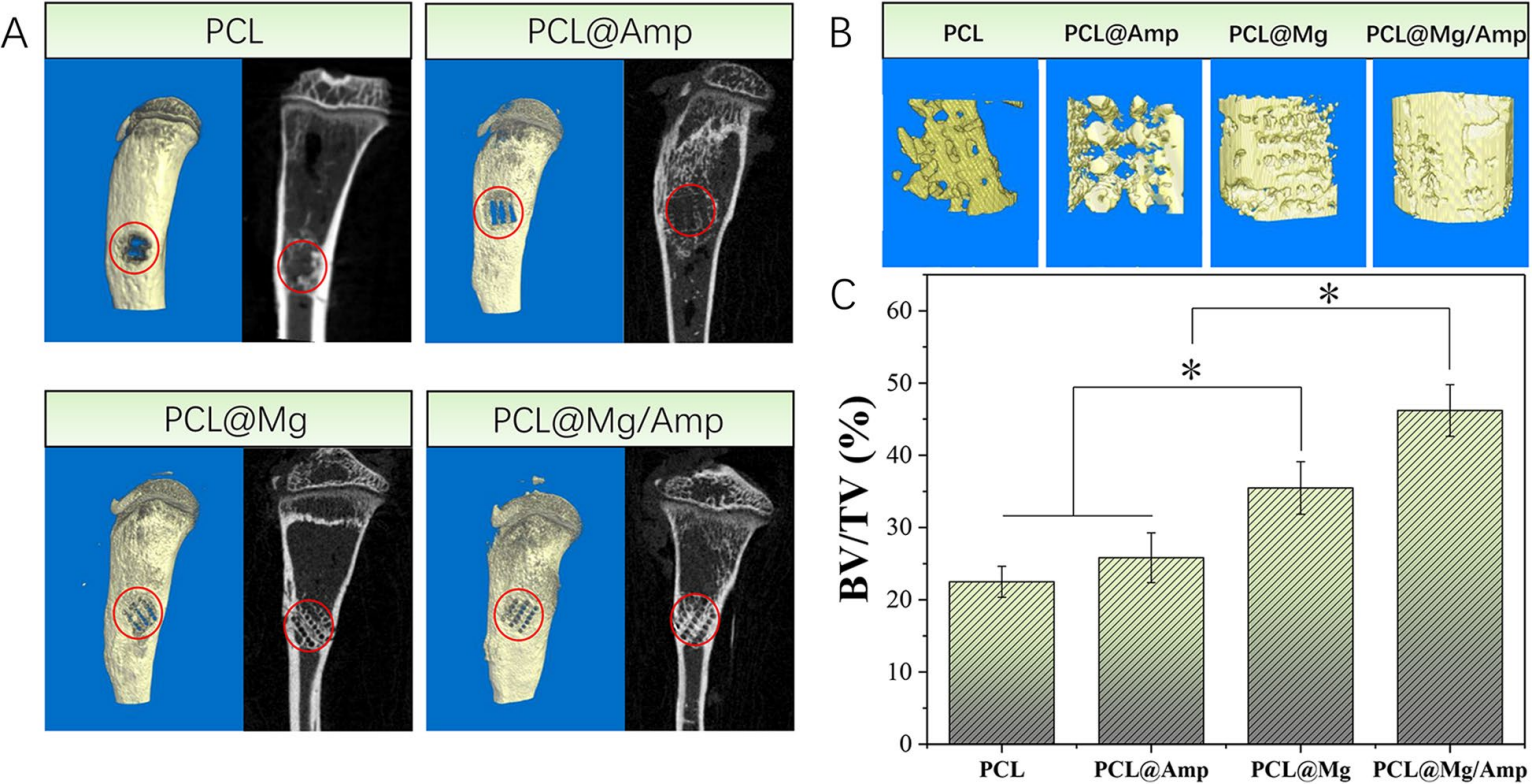
Evaluation of tibial defect repair in rats by micro-CT. **A** Macro CT view and X-ray images, (**B**) 3D view of defect and (**C**) BV/TV assay, *n* = 3 *p* < 0.05)

**Fig. 9:**
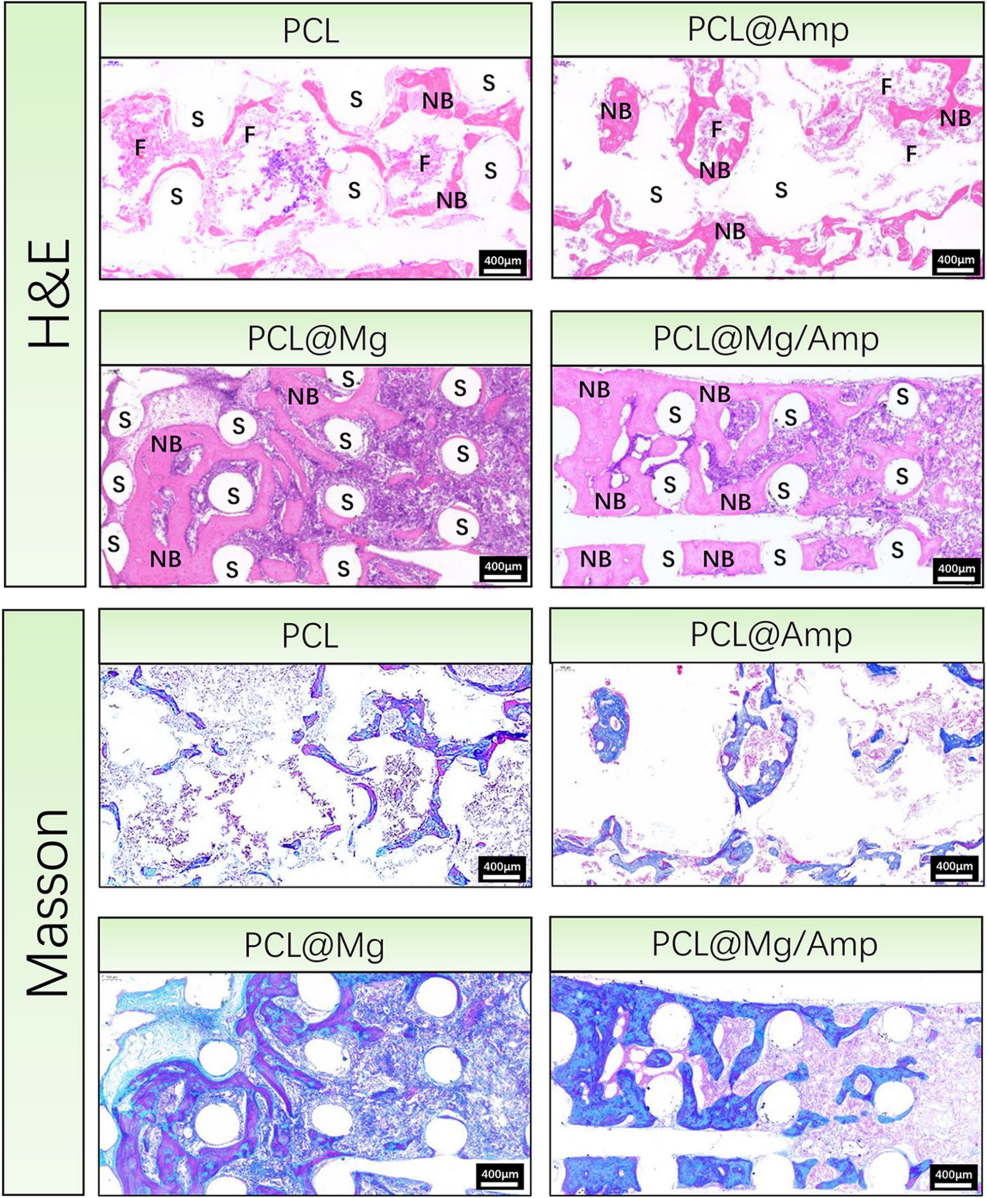
H&E and Masson staining images of tissue slices in tibial defects 8 weeks after surgery. S indicates scaffold, F indicates fibrous tissue, and NB indicates new bone

As shown in Fig. 8, the tibial defect samples were scanned and analysed at 8 weeks after surgery by micro-CT. In the PCL group, only a small amount of new bone was formed along the edge of the defect, and no obvious new bone was formed in the center of the defect. In the PCL@Amp group, a small number of regular porous structures of new bone were observed forming in the center of the defect. In the PCL@Mg and PCL@Mg/Amp groups, the new bone almost completely filled the entire site of the bone defect, and the new bone showed a regular porous structure. In X-ray images, new bone in the PCL@Mg and PCL@Mg/Amp groups can be clearly seen, which is similar to the regular porous structure of the 3D-printed scaffolds. The 3D view images of the defect further proved the repairability effect of different groups of scaffolds on the tibial defect. The BV/TV of the PCL@Mg/Amp (46.20 ± 3.58%) group was significantly higher than that of the PCL@Mg (35.47 ± 3.64%), PCL@Amp (25.82 ± 3.45%) and PCL (22.48 ± 2.15%) groups. As shown in Fig. 9, tissue slices at the tibial defect were analysed by H&E and Masson staining. In the PCL and PCL@Amp groups, a small amount of new bone was formed, and a large amount of fibrous tissue was formed in the interconnected pores of the scaffold, which was not conducive to bone growth and bone healing. In the PCL@Mg and PCL@Mg/Amp groups, the scaffolds were tightly surrounded by a large amount of new bone. The thickness of new bone in the PCL@Mg/Amp group was significantly higher than that in the other groups.

Regeneration of bone defects can be delayed and inhibited by bacterial infection at the defect site. In the clinic, infected bone defects are usually treated by a systemic or local administration of antibiotics to control bacterial infections. This treatment requires additional intervention with antibiotics for the treatment of infected bone defects, which is a time-consuming and tedious procedure [47]. In this study, anti-infective and osteogenic effects were integrated to prepare scaffolds for repairing bone defects. This dual-function in situ bone repair scaffold can release antibiotics at the defect location to play an antibacterial role, and Mg in the scaffold can release Mg^2+^ to promote bone repair. A certain amount of hydrogen gas was also produced during the release of Mg^2+^. If a lot of hydrogen gas was released into the surrounding tissue it could delay bone healing. A large amount of hydrogen gas generated around the scaffold would form gas cavities, and in this study, tissue slices results showed that no gas cavities was formed around the scaffold, which indicated that hydrogen gas was generated in a small amount and fully absorbed by around tiusse [48] (Fig. 9). As shown in Fig. 7, the PCL@Mg/Amp scaffold contained antibiotics, which could reduce bacterial infection at the bone defect site and promote bone repair. Both the PCL@Mg and PCL@Mg/Amp groups contained Mg, which significantly promoted osteogenesis compared with the Mg—free PCL and PCL@Amp groups. These results indicated that the in situ scaffold of PCL@Mg/Amp had dual anti-infection and osteogenesis functions. Although there was still the problem of antibiotic resistance, many antibiotics had been approved by regulatory administration, and the safety *in vivo* was high, so the scaffold containing antibiotics was more easily approved for clinical use [49]. The antibiotic-containing scaffold can reduce the amount and frequency of systemic antibiotic administration in patients. Antimicrobial peptides and AgNPs demonstrate good antimicrobial activity and can treat antimicrobial resistance. However, the *in vivo* safety of antimicrobial peptides and AgNPs is still being evaluated, and intensive studies are needed to prove their *in vivo* safety [50, 51].

## Conclusions

In this study, an in situ PCL bone repair scaffold containing Amp and Mg was prepared using FDM 3D printing technology. The incorporation of Mg and Amp promoted the physical, chemical and biological activities of PCL scaffolds. Mg^2+^ and AMP could be released slowly with scaffold degradation. *In vitro* experiments proved that the Amp component in the scaffold could effectively inhibit the growth of *E. coli* and *S. aureus*. Mg microspheres in scaffolds could release Mg^2+^, which could promote cell adhesion, proliferation and the expression of osteogenic-related genes. *In vivo* experiments showed that the PCL@Mg/Amp scaffold was more effective in repairing tibial defects in rats than the other scaffolds. This dual-function 3D-printed in situ scaffold with antibacterial and osteogenesis properties could be used to treat infected bone defects.

## Data Availability

Not applicable.

## Abbreviations

3D: Three-dimensional
PCL: Polycaprolactone
Amp: Ampicillin
*COL-I*: *collagen I*
mi-RNA: MicroRNA
BioCaP: Biomimetic calcium phosphate
HACC: Hydroxypropyl trimethyl ammonium chitosan
BMP2: Bone morphogenic protein
MRSA: Methicillin-resistant *Staphylococcus aureus*
FDM: Fused deposition modelling
FDA: Food and Drug Administration
pDA: Polydopamine
Mg: Magnesium
*S. aureus*: *Staphylococcus aureus*
*E. coli*: *Escherichia coli*
SLA: Stereo lithography appearance
PLA: Polylactic acid
PLGA: Poly(lactide-co-glycolide)
PDGF: Platelet-derived growth factor
ALP: Alkaline phosphatase
*OPN*: *Osteopontin*
*OCN*: *Osteocalcin*
AgNPs: silver nanoparticles
